# Healthcare access and barriers in Jordan: Insights from a Nationwide Survey

**DOI:** 10.1371/journal.pone.0345456

**Published:** 2026-03-24

**Authors:** Yazan A. Al-Ajlouni, Mohammed Ayyad, Mohammad Tanashat, Omar Al Ta’ani, Hebah AlMomani, Jana Nusier, Mohamed Abouzid, Yazan Nagi, Basile Njei

**Affiliations:** 1 Yale International Medicine Program, Yale University, New Haven, Connecticut, United States of America; 2 Department of Physical Medicine and Rehabilitation, Montefiore Medical Center, Bronx, New York, United States of America; 3 Department of Medicine, Rutgers New Jersey Medical School, Newark, New Jersey, United States of America; 4 Faculty of Medicine, Yarmouk University, Irbid, Jordan; 5 Allegheny Health Network, Pittsburgh, Pennsylvania, United States of America; 6 Faculty of Medicine, Jordan University of Science and Technology, Irbid, Jordan; 7 Doctoral School, Poznan University of Medical Sciences, Poznan, Poland; 8 Department of Physical Pharmacy and Pharmacokinetics, Faculty of Pharmacy, Poznan University of Medical Sciences, Poznan, Poland; 9 SUNY Downstate School of Medicine, Brooklyn, New York, United States of America; 10 Engelhardt School of Global Health and Bioethics, Euclid University, Bangui, Central African Republic and Gambia,; 11 Section of Digestive Diseases, Department of Medicine, Yale University, New Haven, Connecticut, United States of America; 12 VA Connecticut Healthcare, West Haven, Connecticut, United States of America; 13 Ohio University Heritage College of Osteopathic Medicine, Athens, Ohio, United States of America; 14 Yale Liver Center, Yale New Haven Health, New Haven, Connecticut, United States of America; Mutah University, JORDAN

## Abstract

**Background:**

Healthcare access disparities are a significant public health concern, particularly in low- and middle-income countries like Jordan. Despite advancements in healthcare infrastructure, barriers such as financial constraints, cultural norms, and logistical challenges persist, exacerbated by the influx of refugees. This study evaluates the influence of demographic, socioeconomic, and health-related factors on healthcare access and utilization in Jordan, aiming to identify specific barriers and disparities.

**Methods:**

A nationwide survey was conducted in April 2024 using Facebook for recruitment, with 908 eligible participants aged 18–65 completing the survey. Data on healthcare access, barriers, and demographic variables were collected using a validated questionnaire. Statistical analyses included chi-squared tests and Kruskal-Wallis tests to assess differences in healthcare access across subgroups.

**Results:**

Marital status significantly influenced healthcare access, with never-married individuals reporting better access to immediate and specialist care compared to married participants. Employment status revealed disparities, as students had better access to immediate and specialist care, while retired individuals had better access to routine check-ups. Self-reported health status was strongly associated with healthcare access, as individuals reporting excellent health experienced fewer delays and demonstrated higher trust in providers. Barriers to healthcare access included financial constraints, time limitations, and fear of diagnosis, while surgical access challenges centered on cost and waiting times.

**Conclusions:**

Significant disparities in healthcare access exist in Jordan, driven by demographic and socioeconomic factors. Addressing these challenges requires policy reforms, digital health integration, and enhanced public-private partnerships to improve equity and reduce barriers.

## 1. Introduction

Access to healthcare is a fundamental determinant of population health outcomes. Globally, significant disparities persist, with approximately half of the world’s population lacking access to essential health services [1]. This gap is particularly pronounced in low- and middle-income countries, where financial constraints, inadequate infrastructure, and workforce shortages impede healthcare delivery. In 2017, the World Health Organization (WHO) and the World Bank reported that about 100 million people are pushed into extreme poverty annually due to out-of-pocket health expenses [1].

In the Middle East and North Africa (MENA) region, barriers to healthcare access include economic, political, and social dimensions [2]. Political instability and conflict have disrupted healthcare systems, leading to shortages of medical supplies and personnel [3,4]. While contexts vary across the region, several settings illustrate how conflict and displacement have strained both refugee and host populations’ access to care. For instance, in the occupied Palestinian territories, restrictions on movement and systemic discrimination have significantly hindered access to healthcare services. A report by Médecins Sans Frontières highlighted that approximately 300,000 Palestinians in the West Bank face barriers to accessing healthcare, with more than half of the communities lacking adequate services [5]. Similarly, in Lebanon, the Syrian refugee crisis has placed enormous strain on an already fragile healthcare system, resulting in growing unmet health needs among both refugees and host communities [Makhoul et al., 2021; Ammar et al., 2016; Doocy et al., 2016]. These regional parallels highlight how large-scale displacement and limited resources compound access challenges in host countries, including Jordan. Further, economic challenges, such as high unemployment rates and poverty, further exacerbate these barriers, limiting individuals’ ability to afford care [6,7].

The Jordanian healthcare system comprises of public, private and donor-funded sectors which include Ministry of Health (MoH), Royal Medical Services (RMS), university hospitals and United Nations agencies [8]. Despite 70% of the population in Jordan being covered by some form of health insurance disparities in healthcare access remain [9]. Uninsured citizens, low-income workers, and refugees are particularly susceptible to this limited healthcare access. Cultural factors, such as gender norms and societal roles, also influence healthcare-seeking behaviors, potentially delaying timely access to care. Around 28% of total health spending was reported to be out-of-pocket expense which poses a significant financial burden on the population [9].

Furthermore, and as of 2024, approximately 650,000 Syrian refugees have sheltered in Jordan in addition to Palestinian, Iraqi, and other displaced groups [10]. This has substantially affected the healthcare system, increasing public service demand and straining limited resources in underserved areas [11]. This burden has led to overcrowding of healthcare facilities, longer waiting times, and unbalanced distribution of care.

This study aims to examine how demographic factors—including marital status, employment, and self-reported health status—are associated with perceived healthcare access and perceived barriers to medical care among adults in Jordan. By focusing on participants recruited through social media platforms, the study captures perspectives from a broad cross-section of Jordanian society. Understanding these perceptions is essential for developing targeted public health interventions that improve healthcare accessibility and equity. Identifying specific barriers reported by different demographic groups can help policymakers and healthcare providers implement culturally and contextually appropriate strategies to address these challenges and reduce health disparities.

## 2. Methodology

### 2.1. Data and study sample

Participants for this study were recruited using a nationwide survey targeting adults aged 18–65 residing in Jordan at the time of survey administration in April 2024. Facebook was selected as the recruitment platform because of its widespread use in Jordan, with approximately 65.94% of the population actively using the platform during March and April 2024. An advertisement targeting eligible adults aged 18–65 years residing in Jordan was disseminated during this period and managed by an independent researcher. To broaden reach and enhance participant recruitment, snowball sampling was employed by allowing respondents to share the survey link within their social networks. The survey was administered through Qualtrics, a platform for online survey administration previously utilized in health research [12], and was available in both Arabic and English to accommodate the linguistic diversity of participants. The questionnaire was originally developed in English using previously validated survey items. For this study, the survey was translated into Arabic using a forward–backward translation process. Initial translation from English to Arabic was conducted by bilingual members of the study team, followed by back-translation into English by an independent bilingual reviewer. The final Arabic version was reviewed by the study authors to ensure conceptual equivalence, clarity, and cultural appropriateness for the Jordanian context. No substantive modifications were made to the original survey items. Furthermore, and to prevent duplicate responses, IP address restrictions were implemented using the survey platform’s “prevent ballot box stuffing” feature. Additionally, all internal protocol addresses associated with responses were manually reviewed to identify and exclude potential duplicates.

### 2.2. Sample size and completion

A total of 4257 participants accessed the survey link, of which 2434 (57.2%) met eligibility criteria. Reasons for exclusion included being under 18 years of age or residing outside Jordan at the time of the survey. Of the eligible participants, 908 provided complete responses, corresponding to a completion rate of 37.3% among eligible participants and 21.3% of those who initially accessed the survey link.

No a priori formal sample size calculation was performed. Instead, the sample size was determined pragmatically, based on the predefined data collection period in April 2024 and the available resources for Facebook recruitment. All eligible respondents who completed the questionnaire within this period were included, resulting in a final analytic sample of 908 participants.

Although no a priori formal sample size calculation was performed, the achieved sample of 908 participants substantially exceeds the minimum sample sizes typically recommended for detecting small-to-medium effects in cross-sectional analyses, and is comparable to or larger than those used in similar nationwide or online surveys on healthcare access and health-related outcomes in Jordan [13–15].

### 2.3. Measures

#### 2.3.1. Primary measures.

- **Perceived Healthcare Access:** Multiple items were used to evaluate urgent care, primary care, specialty care, and surgical services. Participants were asked to report appointment waiting times, ability to communicate with providers, and relational continuity of care. Example items include: “In the last 12 months, how often did you get an appointment for a check-up or routine care at a doctor’s office or clinic as soon as you needed. Responses were recorded using categorical scales, ranging from “Never” to “Always.”
- **Perceived Barriers to Medical Care:** Barriers were assessed using multiple-choice questions adapted from previously validated instruments (e.g., BRFSS and CAHPS item sets). Participants were asked to select reasons that prevented or delayed medical care from a predefined list (e.g., ‘Too busy to make time,’ ‘Worried about the cost,’ ‘Fear of diagnosis,’ etc.). These responses were used to assess self-reported barriers to accessing care. Perceived barriers to surgical care were similarly assessed through multiple-choice questions, where participants selected from reasons such as financial cost, prolonged waiting times, or lack of service availability
- **Overall Views of the Healthcare System:** Three items were used to evaluate participants’ confidence in the healthcare system’s quality, safety, and affordability. Example: “How confident are you that if you become seriously ill, you will be able to afford the care you need?” Responses ranged from “Very confident” to “Not at all confident.”

#### 2.3.2. Additional measures.

- Sociodemographic: Participants provided information on age, gender (male or female), marital status, employment status (employed, self- employed, unemployed, student or retired), and region of residence within Jordan.

All survey items utilized in this research were adopted from previously validated items [16,17]. While these survey questions were originally developed for use in American populations, they were reviewed by thestudy authors to ensure clarity and contextual appropriateness for the Jordanian population.. No modifications were made to the original survey items. Full survey items relevant to the aims of this study are demonstrated in **Appendix A**.

### 2.4. Statistical analysis

Statistical analyses were conducted using PQStat Software v.1.8.2.238. The Shapiro-Wilk test was applied to evaluate the normality of the data. Normally distributed variables were summarized as mean ± standard deviation (SD), while non-normally distributed data were described using median and interquartile range (IQR). The Mann-Whitney U test and chi-squared (χ2) test were used to compare differences in healthcare access outcomes between male and female participants. To examine differences across employment statuses, the Kruskal-Wallis one-way analysis of variance was employed, followed by post-hoc analysis using Dunn’s Bonferroni correction. A p-value of <0.05 was considered statistically significant for all analyses.

### 2.5. Ethical approval

This study was approved by the University of Jordan Ethical Committee on March 18th, 2024 (Decision No. 181/2024). Participation was voluntary, and all responses were anonymized to ensure confidentiality. Electronic informed consent was obtained from all participants prior to participation. The first page of the Qualtrics survey presented an information sheet detailing the study purpose, procedures, risks, benefits, and confidentiality data. Participants were required to actively indicate their consent by selecting “I agree to participate” before accessing any survey questions; respondents who did not provide consent were automatically exited from the survey. Only adults aged 18–65 years were eligible to participate; no minors were included in this study.

## 3. Results

### 3.1. Demographic characteristics

We included 908 participants with an average age of 31.4 ± 13.79. The sex distribution shows that 55.6% of the participants are female and 44.4% are male. In terms of employment, most of the participants are students (49.8%), followed by employed individuals (31.6%), unemployed individuals (11.2%), and retired individuals (7.4%). As for marital status, most of the participants have never been married (62%), while 32.6% are married, and the rest are either separated (1.4%), widowed (2.3%), or divorced (1.7%). Approximately 50% of participants had very good health status as perceptually reported. The participants are from various cities, with the majority residing in Irbid (45.4%) and Balqa (38%). Table 1 summarizes the distribution of participants across cities in Jordan.

**Table 1:** Demographic characters of the participants, frequencies reported as N (%).

Age	31.43 ± 13.79; [24 (22 - 41)]^*^
**Sex** *Female* *Male*	505 (55.6) 403 (44.4)
**Employment** *Retired* *Student* *Unemployed* *Employed*	67 (7.4) 452 (49.8) 102 (11.2) 287 (31.6)
**Marital Status** *Never married* *Separated* *Widowed* *Divorced* *Married*	563 (62) 13 (1.4) 21 (2.3) 15 (1.7) 296 (32.6)
**City** *Karak* *Madaba* *Zarqa* *Amman* *Balqa* *Mafraq* *Jerash* *Ajloun* *Aqaba* *Ma'an* *Tafila* *Irbid*	3 (0.3) 12 (1.3) 5 (0.6) 26 (2.9) 345 (38) 22 (2.4) 6 (0.6) 21 (2.3) 15 (1.6) 39 (4.3) 2 (0.2) 412 (45.4)
**Reported Health Status** *Excellent* *Very good* *Good* *Fair* *Poor* *Not sure*	190 (20.9) 439 (48.3) 209 (23.0) 58 (6.4) 9 (1.0) 3 (0.3)

* reported as mean ± SD; [median (IQR)]

### 3.2. Sex

No significant differences were observed between males and females regarding any of health access dimensions (immediate care, routine check-ups, specialist appointments, communication with doctors and trust in healthcare providers) (Table 2).

**Table 2:** Differences between sex and healthcare access.

Answers	Female	Male	Chi-squared test	
			χ2 (df)	Sig.
In the last 12 months, how many days did you usually have to wait for an appointment when you needed care right away				
*Did not need an appointment*	147 (29.1)	120 (29.8)	5.4 (5)	0.370
*more than 7 days*	62 (12.3)	52 (12.9)		
*4-7 days*	29 (5.7)	24 (6)		
*2-3 days*	71 (14.1)	45 (11.2)		
*1 day*	54 (10.7)	60 (14.9)		
*Same day*	142 (28.1)	102 (25.3)		
In the last 12 months, how often did you get an appointment for a check-up or routine care at a doctor’s office or clinic as soon as you needed?				
*Did not need an appointment*	84 (16.6)	79 (19.6)	5.5 (4)	0.244
*Always*	58 (11.5)	31 (7.7)		
*Usually*	74 (14.7)	62 (15.4)		
*Sometimes*	221 (43.8)	185 (45.9)		
*Never*	68 (13.5)	46 (11.4)		
In the last 12 months, how often did you get an appointment to see a specialist as soon as you needed?				
*Did not need an appointment*	89 (17.6)	87 (21.6)	3.0 (4)	0.555
*Always*	59 (11.7)	41 (10.2)		
*Usually*	78 (15.4)	66 (16.4)		
*Sometimes*	215 (42.6)	164 (40.7)		
*Never*	64 (12.7)	45 (11.2)		
Thinking about your regular doctor, can you communicate with them?				
*No*	94 (18.6)	86 (21.3)	1.2 (4)	0.559
*Maybe*	145 (28.7)	108 (26.8)		
*Yes*	266 (52.7)	209 (51.9)		
In the last 12 months, did you feel you could trust this provider with your medical care?				
*No*	67 (13.3)	67 (16.6)	2.3 (4)	0.315
*Maybe*	102 (20.2)	84 (20.8)		
*Yes*	336 (66.5)	252 (62.5)		

### 3.3. Marital status

The results from Table 3 indicate significant differences in healthcare access based on marital status. For immediate care, there was a significant difference (χ² = 38.4, df = 20, p = 0.008), with never married individuals generally reporting higher access and less need for medical appointments compared to married individuals, as highlighted by the post-hoc analysis (p = 0.015). Moreover, significant differences were observed for specialist appointments (χ² = 41.1, df = 16, p = 0.001), with never married individuals again reporting higher access and less need for specialist appointments, supported by the post-hoc analysis (p = 0.0001). Communication with regular doctors differed across marital status groups (χ² = 15.0, df = 8, p = 0.059), with married individuals more likely to communicate effectively with their doctors (p = 0.028). No significant differences were found for routine check-ups, and trust in healthcare providers did not show significant differences.

**Table 3:** Differences between marital status and healthcare access.

Answers	Never married	Separated	Widowed	Divorced	Married	Chi-squared test	
						χ2 (df)	Sig.
In the last 12 months, how many days did you usually have to wait for an appointment when you needed care right away							
*Did not need an appointment*	180 (32)	3 (23.1)	7 (33.3)	3 (20)	74 (25)	38.4 (20)	0.008†
*more than 7 days*	59 (10.5)	0 (0)	2 (9.5)	3 (20)	50 (16.9)		
*4-7 days*	24 (4.3)	3 (23.1)	3 (14.3)	1 (6.7)	22 (7.4)		
*2-3 days*	62 (11)	3 (23.1)	4 (19)	2 (13.3)	45 (15.2)		
*1 day*	70 (12.4)	3 (23.1)	1 (4.8)	2 (13.3)	38 (12.8)		
*Same day*	168 (29.8)	1 (7.7)	4 (19)	4 (26.7)	67 (22.6)		
In the last 12 months, how often did you get an appointment for a check-up or routine care at a doctor’s office or clinic as soon as you needed?							
*Did not need an appointment*	106 (18.8)	1 (7.7)	4 (19)	3 (20)	49 (16.6)	19.2 (16)	0.259
*Always*	46 (8.2)	3 (23.1)	3 (14.3)	1 (6.7)	36 (12.2)		
*Usually*	87 (15.5)	2 (15.4)	2 (9.5)	2 (13.3)	43 (14.5)		
*Sometimes*	241 (42.8)	6 (46.2)	8 (38.1)	6 (40)	145 (49)		
*Never*	83 (14.7)	1 (7.7)	4 (19)	3 (20)	23 (7.8)		
In the last 12 months, how often did you get an appointment to see a specialist as soon as you needed?							
*Did not need an appointment*	124 (22)	0 (0)	2 (9.5)	3 (20)	47 (15.9)	41.1 (16)	0.001††
*Always*	60 (10.7)	3 (23.1)	2 (9.5)	3 (20)	32 (10.8)		
*Usually*	95 (16.9)	1 (7.7)	4 (19)	1 (6.7)	43 (14.5)		
*Sometimes*	204 (36.2)	6 (46.2)	8 (38.1)	5 (33.3)	156 (52.7)		
*Never*	80 (14.2)	3 (23.1)	5 (23.8)	3 (20)	18 (6.1)		
Thinking about your regular doctor, can you communicate with them?							
*No*	127 (22.6)	4 (30.8)	4 (19)	5 (33.3)	40 (13.5)	15.0 (8)	0.059†††
*Maybe*	160 (28.4)	2 (15.4)	6 (28.6)	3 (20)	82 (27.7)		
*Yes*	276 (49)	7 (53.8)	11 (52.4)	7 (46.7)	174 (58.8)		
In the last 12 months, did you feel you could trust this provider with your medical care?							
*No*	87 (15.5)	3 (23.1)	2 (9.5)	2 (13.3)	40 (13.5)	6.5 (8)	0.592
*Maybe*	117 (20.8)	5 (38.5)	5 (23.8)	4 (26.7)	55 (18.6)		
*Yes*	359 (63.8)	5 (38.5)	14 (66.7)	9 (60)	201 (67.9)		

† Significant differences married and never married [p = 0.015, post-hoc (Multiple comp. Bonferroni)]

†† Significant differences married and never married [p = 0.0001, post-hoc (Multiple comp. Bonferroni)]

††† Significant differences married and never married [p = 0.028, post-hoc (Multiple comp. Bonferroni)]

### 3.4. Employment

Overall, students generally had higher access to immediate care and specialist appointments, while retired individuals had higher access to routine check-ups, and employed individuals reported better communication with their regular doctors (Table 4). For immediate care, there was a significant difference (χ² = 27.9, df = 15, p = 0.022), with students reporting higher access and not needing an appointment compared to retired individuals, as highlighted by the post-hoc analysis (p = 0.03). Significant differences were also observed for routine check-ups (χ² = 26.6, df = 12, p = 0.009), with retired individuals reporting higher access compared to students, as they reported higher number of appointments compared to students (p = 0.007). For specialist appointments there was a significant difference (χ² = 24.7, df = 12, p = 0.017). A higher proportion of students (22.8%) reported not needing an appointment compared to employed individuals (16.7%). However, when appointments were needed, students reported higher access compared to employed individuals (p = 0.035). Communication with regular doctors showed a significant difference (χ² = 16.9, df = 6, p = 0.010), with employed individuals having a higher proportion reporting being able to communicate effectively with their doctors compared to students (p = 0.006). No significant differences were found in trust in healthcare providers (χ² = 2.4, df = 6, p = 0.878).

**Table 4:** Differences between employment status and healthcare access.

Answers	Retired	Student	Unemployed	Employed	Chi-squared test	
					χ2 (df)	Sig.
In the last 12 months, how many days did you usually have to wait for an appointment when you needed care right away						
*Did not need an appointment*	10 (14.9)	149 (33)	33 (32.4)	75 (26.1)	27.9 (15)	0.022†
*more than 7 days*	16 (23.9)	50 (11.1)	10 (9.8)	38 (13.2)		
*4-7 days*	4 (6)	22 (4.9)	8 (7.8)	19 (6.6)		
*2-3 days*	12 (17.9)	51 (11.3)	12 (11.8)	41 (14.3)		
*1 day*	5 (7.5)	50 (11.1)	13 (12.7)	46 (16)		
*Same day*	20 (29.9)	130 (28.8)	26 (25.5)	68 (23.7)		
In the last 12 months, how often did you get an appointment for a check-up or routine care at a doctor’s office or clinic as soon as you needed?						
*Did not need an appointment*	9 (13.4)	89 (19.7)	19 (18.6)	46 (16)	26.6 (12)	0.009††
*Always*	15 (22.4)	33 (7.3)	10 (9.8)	31 (10.8)		
*Usually*	10 (14.9)	72 (15.9)	19 (18.6)	35 (12.2)		
*Sometimes*	28 (41.8)	190 (42)	44 (43.1)	144 (50.2)		
*Never*	5 (7.5)	68 (15)	10 (9.8)	31 (10.8)		
In the last 12 months, how often did you get an appointment to see a specialist as soon as you needed?						
*Did not need an appointment*	8 (11.9)	103 (22.8)	17 (16.7)	48 (16.7)	24.7 (12)	0.017†††
*Always*	12 (17.9)	43 (9.5)	11 (10.8)	34 (11.8)		
*Usually*	9 (13.4)	71 (15.7)	17 (16.7)	47 (16.4)		
*Sometimes*	34 (50.7)	167 (36.9)	44 (43.1)	134 (46.7)		
*Never*	4 (6)	68 (15)	13 (12.7)	24 (8.4)		
Thinking about your regular doctor, can you communicate with them?						
*No*	13 (19.4)	108 (23.9)	19 (18.6)	40 (13.9)	16.9 (6)	0.010††††
*Maybe*	13 (19.4)	132 (29.2)	30 (29.4)	78 (27.2)		
*Yes*	41 (61.2)	212 (46.9)	53 (52)	169 (58.9)		
In the last 12 months, did you feel you could trust this provider with your medical care?						
*No*	11 (16.4)	68 (15)	15 (14.7)	40 (13.9)	2.4 (6)	0.878
*Maybe*	13 (19.4)	95 (21)	25 (24.5)	53 (18.5)		
*Yes*	43 (64.2)	289 (63.9)	62 (60.8)	194 (67.6)		

† Significant differences between student and retired [p = 0.03, post-hoc (Multiple comp. Bonferroni)]

†† Significant differences between student and retired [p = 0.007, post-hoc (Multiple comp. Bonferroni)]

††† Significant differences between student and employed [p = 0.035, post-hoc (Multiple comp. Bonferroni)]

†††† Significant differences between student and employed [p = 0.006, post-hoc (Multiple comp. Bonferroni)]

### 3.5. Health status

Overall, individuals in better health statuses generally had higher access to immediate care, routine check-ups, specialist appointments, and a higher proportion reported effective communication and trust with their healthcare providers as compared to other groups. (Table 5). For immediate care, there was a significant difference (χ² = 82.8, df = 25, p < 0.0001), with individuals reporting “Excellent” health having the highest percentage of not needing an appointment (42.1%) and those in “Poor” health having the highest percentage of waiting more than 7 days (33.3%). Significant differences were also observed for routine check-ups (χ² = 62.6, df = 20, p < 0.0001), with individuals in “Excellent” health reporting the highest proportion of not needing an appointment (23.7%) and those in “Poor” health reporting not getting an appointment as soon as needed (33.3%). For specialist appointments, there was a significant difference (χ² = 52.8, df = 20, p < 0.0001), with 24.7% of individuals with “Excellent” health reported not requiring an appointment and 44.4% of individuals with “Poor” health reported to wait more than 7 days. Communication with regular doctors showed a significant difference (χ² = 27.7, df = 10, p = 0.002), with individuals in “Very Good” health had the highest proportion reporting effective communication with their doctors (57.2%) compared to those in “Fair” health (41.4%). Trust in healthcare providers also showed a significant difference (χ² = 19.6, df = 10, p = 0.003), with individuals in “Excellent” health having the highest proportion reporting trust in their health providers (66.3%) compared to those in “Fair” health (51.7%).

**Table 5:** Differences between health status and healthcare access.

Answers	I am not sure	Poor	Fair	Good	Very Good	Excellent	Chi-squared test	
							χ2 (df)	Sig.
**In the last 12 months, how many days did you usually have to wait for an appointment when you needed care right away**								
*Did not need an appointment*	0 (0)	2 (22.2)	6 (10.3)	45 (21.5)	134 (30.5)	80 (42.1)	82.8 (25)	<0.0001†
*more than 7 days*	0 (0)	3 (33.3)	15 (25.9)	33 (15.8)	49 (11.2)	14 (7.4)		
*4-7 days*	0 (0)	0 (0)	9 (15.5)	18 (8.6)	18 (4.1)	8 (4.2)		
*2-3 days*	1 (33.3)	0 (0)	7 (12.1)	39 (18.7)	52 (11.8)	17 (8.9)		
*1 day*	2 (66.7)	1 (11.1)	4 (6.9)	30 (14.4)	59 (13.4)	18 (9.5)		
*Same day*	0 (0)	3 (33.3)	17 (29.3)	44 (21.1)	127 (28.9)	53 (27.9)		
**In the last 12 months, how often did you get an appointment for a check-up or routine care at a doctor’s office or clinic as soon as you needed?**								
*Did not need an appointment*	1 (33.3)	1 (11.1)	1 (1.7)	23 (11)	92 (21)	45 (23.7)	62.6 (20)	<0.0001††
*Always*	1 (33.3)	1 (11.1)	7 (12.1)	27 (12.9)	43 (9.8)	10 (5.3)		
*Usually*	1 (33.3)	1 (11.1)	17 (29.3)	39 (18.7)	58 (13.2)	20 (10.5)		
*Sometimes*	0 (0)	3 (33.3)	27 (46.6)	95 (45.5)	205 (46.7)	76 (40)		
*Never*	0 (0)	3 (33.3)	6 (10.3)	25 (12)	41 (9.3)	39 (20.5)		
**In the last 12 months, how often did you get an appointment to see a specialist as soon as you needed?**								
*Did not need an appointment*	0 (0)	1 (11.1)	3 (5.2)	29 (13.9)	96 (21.9)	47 (24.7)	52.8 (20)	<0.0001†††
*Always*	1 (33.3)	1 (11.1)	4 (6.9)	27 (12.9)	50 (11.4)	17 (8.9)		
*Usually*	1 (33.3)	2 (22.2)	20 (34.5)	46 (22)	58 (13.2)	17 (8.9)		
*Sometimes*	0 (0)	4 (44.4)	27 (46.6)	85 (40.7)	186 (42.4)	77 (40.5)		
*Never*	1 (33.3)	1 (11.1)	4 (6.9)	22 (10.5)	49 (11.2)	32 (16.8)		
**Thinking about your regular doctor, can you communicate with them?**								
*No*	0 (0)	0 (0)	11 (19)	47 (22.5)	81 (18.5)	41 (21.6)	27.7 (10)	0.002††††
*Maybe*	2 (66.7)	5 (55.6)	23 (39.7)	74 (35.4)	107 (24.4)	42 (22.1)		
*Yes*	1 (33.3)	4 (44.4)	24 (41.4)	88 (42.1)	251 (57.2)	107 (56.3)		
**In the last 12 months, did you feel you could trust this provider with your medical care?**								
*No*	0 (0)	2 (22.2)	11 (19)	41 (19.6)	52 (11.8)	28 (14.7)	19.6 (10)	0.003†††††
*Maybe*	1 (33.3)	3 (33.3)	17 (29.3)	50 (23.9)	79 (18)	36 (18.9)		
*Yes*	2 (66.7)	4 (44.4)	30 (51.7)	118 (56.5)	308 (70.2)	126 (66.3)		

† Significant differences between (i) fair and very good [p = 0.03]; (ii) fair and excellent (p < 0.001); (iii) good and very good (p = 0.02); and (iv) good and exellent (p < 0.001) [post-hoc (Multiple comp. Bonferroni)]

†† Significant differences between (i) fair and very good [p = 0.01]; (ii) fair and excellent (p < 0.001); (iii) good and excellent (p < 0.001); and (iv) very good and exellent (p = 0.012) [post-hoc (Multiple comp. Bonferroni)]

††† Significant differences between (i) fair and very good [p = 0.001]; (ii) fair and excellent (p < 0.001); and (iii) good and excellent (p = 0.004)

†††† Significant differences between good and very good [p = 0.018, post-hoc (Multiple comp. Bonferroni)]

††††† Significant differences between good and very good [p = 0.029, post-hoc (Multiple comp. Bonferroni)]

### 3.6. Barries *to* healthcare access and surgery

**Fig 1:**
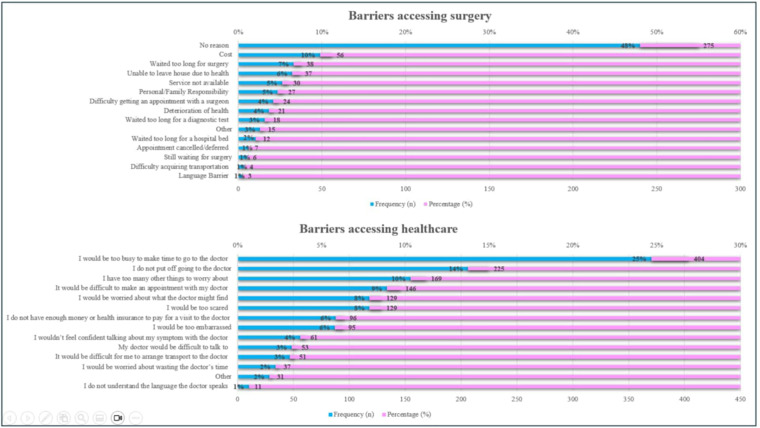
Reported barriers to accessing healthcare services and surgical care. Bar charts display the frequency (n) and percentage (%) of participants reporting barriers to accessing surgery (top panel) and general healthcare services (bottom panel), including financial, logistical, and communication-related challenges.

Regarding access to surgery, the most commonly reported response (48%) was that participants reported no reason for being unable to access surgical care. Other notable reasons include waiting too long for surgery (7%), being unable to leave the house due to health issues (6%), and the unavailability of services (5%). Personal or family responsibilities also impact 5% of the individuals. Concerning the barriers to healthcare access, the most common reason, affecting 25% of respondents, is being too busy to make time for a visit. Following this, 14% of individuals do not put off going to the doctor. Other notable reasons include having too many other things to worry about (10%), difficulty making an appointment (9%), and fear of what the doctor might find (8%). Financial constraints and embarrassment each affect 6% of respondents. Descriptive data for perceived barriers to healthcare access and surgical care were summarized using frequencies and percentages and presented graphically (Fig 1**).**

## 4. Discussion

This study sought to investigate the association between demographic, socioeconomic, and health-related factors on healthcare access and utilization among Jordanian adults, aiming to identify specific barriers and disparities. Our study highlights significant disparities in healthcare access in Jordan, driven by demographic and socioeconomic factors. Notably, marital status influenced access, with never-married individuals reporting better access to immediate care and specialist appointments than married participants. Employment status also played a critical role, as students exhibited superior access to immediate and specialist care, while retired individuals had better access to routine check-ups. Self-reported health status emerged as a strong determinant, with individuals in better health experiencing fewer delays and reporting higher trust in healthcare providers compared to those in poor health. Additionally, barriers to surgery were primarily related to cost, waiting times, and health-related immobility, while general barriers to healthcare access included being too busy and fear of diagnosis.

Marital status significantly influenced access, with never-married individuals reporting better access to immediate and specialist care compared to their married counterparts. For instance, only 16.9% of married participants accessed immediate care when needed, compared to 29.8% of never-married individuals. This trend mirrors findings in the MENA region and developed countries, where social roles and family responsibilities often hinder healthcare-seeking behaviors among married individuals [18,19]. Additionally, a higher proportion of married participants reported being able to communicate with their primary physician compared to never-married participants. Interestingly, gender did not emerge as a significant determinant of healthcare access in our study. This contrasts with two review articles assessing gender variation in health utilization, which showed findings on gender disparities in both the Arab world and globally, where women often face barriers due to cultural preferences for same-gender providers and logistical challenges [19,20]. Conversely, our findings revealed equal proportions of men (25.3%) and women (28.1%) reporting same-day access to immediate care. This may reflect improvements in gender equity within Jordan’s healthcare system. For example, Jordan’s National Strategy for Women (2020–2025) focuses on health equity emphasizing women’s access to quality healthcare and gender-responsive planning [21]. These initiatives with increased healthcare literacy can contribute to better equitable healthcare across genders.

Employment status emerged as a significant factor influencing healthcare access. Students reported superior access to immediate and specialist care, with 33% not requiring an appointment for immediate care and 22.8% bypassing the need for specialist visits. In contrast, retired individuals had the highest access to routine check-ups, with 22.4% reporting timely appointments compared to 10.8% of employed individuals. These findings align with studies in the MENA region on barriers to early presentation to colorectal cancer for instance, where a cross-sectional study in Palestine showed that although employment could provide a financial buffer, it limits the time available for healthcare utilization which might affect the perception of individuals about access to healthcare for their medical concerns [22]. Moreover, the observed patterns suggest that younger populations are less reliant on structured healthcare services due to employment related factors including time restrictions and limited availability, while retired individuals prioritize routine and preventive care measures possibly due to more time availability. Policymakers should consider these trends to design flexible healthcare systems accommodating diverse employment statuses.

Moreover, participants’ self-reported health status significantly influenced healthcare access, mirroring global trends linking better overall health with improved utilization of healthcare services. Individuals in excellent health were significantly less likely to require immediate care, with 42.1% not needing appointments compared to only 22.2% in poor health. Similarly, 44.4% of participants reporting poor health waited more than seven days for a specialist appointment, showing the complexity and urgency of needs of the patient simultaneously highlighting the limited availability of services for specialized care in public healthcare. Similar findings have been reported in studies on publicly insured populations like those under Medicaid in the U.S., where structural inefficiencies lead to delay in access to care. While this study did not collect data on income, the comparison is made to represent the similarity in system-level limitations rather than income-related differences [23]. The significant association between health status and trust in providers, with 66.3% of individuals in excellent health trusting their providers compared to 51.7% in fair health, further indicates the reciprocal relationship between healthcare experiences and perceptions of quality [24,25].

Importantly, surgical access in Jordan presents a microcosm of broader healthcare challenges. While nearly half of respondents reported no barriers to surgery, financial constraints affected 10%, and 7% faced excessive waiting times. Health-related immobility and unavailable services were additional barriers. Compared to broader regional findings, these rates are relatively moderate, potentially reflecting Jordan’s public health investments [26]. However, the persistence of logistical and systemic issues highlights the need for capacity-building and infrastructure enhancements. General barriers to healthcare access included being too busy to make an appointment and fear of diagnosis, echoing cultural and psychological hurdles documented in the Arab Barometer survey [26,27]. These barriers were more pronounced among younger populations. Furthermore, Jordan’s healthcare access challenges mirror those documented in neighboring MENA countries, with urban-rural disparities, logistical barriers, and cultural influences playing central roles. For instance, several studies highlight the role of transportation costs and urban concentration of healthcare providers in limiting access [28,29]. Moreover, the persistent role of financial barriers in surgical access aligns with findings from Tunisia and Morocco, where out-of-pocket costs remain a major deterrent to healthcare utilization [19]. System-level barriers, such as waiting times and appointment availability, also parallel global trends in barriers to healthcare access [23,30,31]. For instance, Medicaid populations in the US face similar delays in accessing preventive care despite insurance coverage [23]. This indicates that financial interventions alone are insufficient and must be complemented by systemic reforms, including expanded provider capacity, reduced waiting times, and improved care coordination, which remain persistent barriers to healthcare access across both high- and middle-income countries. [32–34]. Thus, addressing these challenges in Jordan require a multifaceted approach combining financial support with systemic reforms to improve availability of serviced, responsiveness, and continuity of care.

### 4.1. Strengths and limitations

This study provides a comprehensive and localized exploration of healthcare access disparities in Jordan, highlighting how demographic and socioeconomic factors influence healthcare utilization. One of the strengths of this research is its ability to combine multiple determinants—such as marital status, employment, and self-reported health status—into a single analysis. This approach offers a holistic understanding of how various factors intersect to shape individuals’ experiences with healthcare services. Moreover, the comparative analysis with findings from other MENA countries and the U.S. helps contextualize the results within global healthcare access trends, further emphasizing the relevance of these issues in Jordan and the broader region. Another key strength is the policy relevance of the findings. By identifying specific barriers (e.g., cost, waiting times, and fear of diagnosis), the study provides actionable recommendations that align with current healthcare challenges in Jordan. The potential for future interventions, such as the integration of telemedicine and expansion of public-private partnerships, can guide policymakers in addressing gaps in healthcare access and improving service delivery for diverse populations.

Despite its strengths, this study has several limitations that should be considered when interpreting the findings. First, the cross-sectional design limits the ability to draw causal inferences, and the observed associations between demographic characteristics, self-reported health status, and healthcare access should be interpreted descriptively rather than causally. In addition, the analyses were primarily bivariate and did not account for potential confounding or interrelationships between factors such as age, marital status, employment status, and health status. Future studies incorporating multivariable models would help clarify the independent contribution of these factors.

Second, the study relied on self-reported data, which may be subject to recall bias and social desirability bias. Moreover, several key constructs, including trust in healthcare providers, fear of diagnosis, and perceived barriers to care, are inherently subjective and may not fully align with objectively measured system-level barriers. Although survey items were adapted from widely used and previously validated U.S.-based instruments and underwent forward–backward translation and expert review, no formal cultural or psychometric validation was conducted for the Jordanian context. This may affect the interpretation and cross-cultural comparability of subjective measures.

Third, the representativeness of the sample may be limited. Recruitment via social media, particularly Facebook, facilitated broad and rapid distribution but may have excluded individuals with limited internet access, lower digital literacy, or older age, introducing potential selection bias. Additionally, the sample was weighted toward women, students, and never-married individuals, potentially underrepresenting older adults, individuals from lower socioeconomic backgrounds, refugees, and those with chronic health conditions.

Finally, the questionnaire was not formally piloted prior to full deployment. Although all items were derived from previously validated instruments and reviewed for clarity and contextual relevance, the absence of pilot testing may have limited the identification of minor issues related to question interpretation or response burden.

Future research should aim for a larger, more diverse sample to better capture the full spectrum of healthcare access issues across Jordan. Finally, while the study explores several key factors influencing healthcare access, it does not fully delve into the cultural and psychological barriers that may affect healthcare-seeking behavior. Certain structural barriers like disability status, caregiving burden etc. have also not been accounted for which are highlighted in regional studies. Qualitative research focusing on individuals’ attitudes, beliefs, and experiences with the healthcare system could provide a deeper understanding of these barriers and further inform targeted interventions.

### 4.2. Future directions and policy implications

Addressing the barriers to healthcare access in Jordan requires a multifaceted approach that combines policy reform, community engagement, and technological utilization. Future efforts should prioritize the integration of digital health solutions such as telemedicine and mobile health platforms to improve accessibility and reduce logistical barriers, particularly for individuals in remote or underserved areas. Use of telemedicine and mobile health services showed promising improvement in healthcare accessibility in the MENA region especially during and after the COVID-19 pandemic providing substantial evidence for importance of integration of digital health tools [35,36].

Additionally, fostering public-private partnerships (PPPs) can help expand healthcare infrastructure and increase the availability of specialist services and reasonable appointment times, reducing the strain on public healthcare systems. Jordan has previously benefited from PPPs in health, profoundly in tertiary care expansion [37]. To better understand and address cultural and psychological barriers, qualitative research exploring patient attitudes and beliefs about healthcare, especially among underserved groups, is essential.

Policymakers should also consider implementing health equity monitoring frameworks to track progress and identify gaps in service delivery. Finally, education and training programs for healthcare providers should emphasize cultural competency and patient-centered care to build trust and improve communication, ultimately enhancing patient experiences and outcomes in Jordan [38].

## 5. Conclusion(s)

This study exemplifies the significant disparities in healthcare access in Jordan, highlighting the influence of demographic, socioeconomic, and health-related factors on individuals’ ability to access care. Key determinants, such as marital status, employment, and self-reported health status, reveal distinct patterns that suggest targeted interventions are needed to address these disparities. While barriers like cost, waiting times, and fear of diagnosis remain persistent challenges, the findings emphasize the potential for policy reform, the integration of digital health solutions, and enhanced public-private partnerships to improve access, especially for underserved populations. Ultimately, addressing these barriers will require a multifaceted approach that combines systemic improvements with cultural sensitivity and community engagement to ensure equitable healthcare access for all Jordanians.

## Supporting information

S1 File Survey questionnaire used in the study. The questionnaire included items assessing self-reported health status, healthcare access (urgent care, primary care, specialty care, communication access, and relational continuity), barriers to medical care, perceptions and access to surgical care, and demographic characteristics of participants.
(DOCX)

S1 Fig Graphical Abstract. (PNG)
