# Supplementary material for: Healthcare access and barriers in Jordan: Insights from a Nationwide Survey
Source: PLoS One. 2026 Mar 24;21(3):e0345456. doi: 10.1371/journal.pone.0345456 (PMC13012521; doi:10.1371/journal.pone.0345456)
Supplement: S1 File — The questionnaire included items assessing self-reported health status, healthcare access (urgent care, primary care, specialty care, communication access, and relational continuity), barriers to medical care, perceptions and access to surgical care, and demographic characteristics of participants. (DOCX) [file pone.0345456.s001.docx]

**S1 File.** Survey questionnaire used in the study. The questionnaire included items assessing self-reported health status, healthcare access (urgent care, primary care, specialty care, communication access, and relational continuity), barriers to medical care, perceptions and access to surgical care, and demographic characteristics of participants.

**S1 File**

**Health Status**

Would you say that in general your health is—

1. Excellent
2. Very Good
3. Good
4. Fair
5. Poor
6. Don’t know/Not sure

Variable Code: HSTATUS

Origin: *Centers for Disease Control and Prevention (CDC). Behavioral Risk Factor Surveillance System Survey Questionnaire. Atlanta, Georgia: U.S. Department of Health and Human Services, Centers for Disease Control and Prevention, [2020]*

**Health Care Access**

1. **Urgent Care Access**

In the last 12 months, how many days did you usually have to wait for an appointment when you needed care right away?

1. Same day
2. 1 day
3. 2 to 3 days
4. 4 to 7 days
5. More than 7 days

Variable Code: UCACCESS

Origin: CAHPS-HP

1. **Primary Care Access**

In the last 12 months, how often did you get an appointment for a check-up or routine care at a doctor’s office or clinic as soon as you needed?

1. Never
2. Sometimes
3. Usually
4. Always

Variable Code: PCACCESS

Origin: CAHPS-HP

1. **Specialty Care Access**

In the last 12 months, how often did you get an appointment to see a specialist as soon as you needed?

1. Never
2. Sometimes
3. Usually
4. Always

Variable Code: SCACCESS

Origin: CAHPS-HP

1. **Communication Access Between Visits**

Thinking about your regular doctor, can you communicate them?

1. Yes
2. No
3. Don’t know

Variable Code: CACCESS

Origin: *The Commonwealth Fund (CMWF) International Health Policy Survey in Seven Countries (2007) http://www.commonwealthfund.org/surveys/surveys_show.htm?doc_id=568326 Accessed January 25th, 2022*

1. **Relational Continuity**

In the last 12 months, did you feel you could trust this provider with your medical care?

1. Yes
2. No
3. Don’t know

Variable Code: RCONT

Origin: *Weech-Maldonado, Robert, et al. "The Consumer Assessment of Healthcare Providers and Systems (CAHPS®) Cultural Competence (CC) Item Set." Medical care 50.9 0 2 (2012): S22.*

**Barriers to Medical Care**

Sometimes people put off going to see the doctor, even when they have a symptom that they think might be serious. Could you say if any of these might put you off going to the doctor?

1. I would be too embarrassed
2. I would be too scared
3. I would be worried about wasting the doctor’s time
4. My doctor would be difficult to talk to
5. It would be difficult to make an appointment with my doctor
6. I would be too busy to make time to go to the doctor
7. I have too many other things to worry about
8. It would be difficult for me to arrange transport to the doctor
9. I would be worried about what the doctor might find
10. I wouldn’t feel confident talking about my symptom with the doctor
11. I do not have enough money or health insurance to pay for a visit to the doctor
12. I do not understand the language the doctor speaks
13. Are there any other reasons you may not visit a doctor? (Please specify below)

Variable Code: HBARRI

*Surgical Care Access*

1. Have you ever sought surgical care or undergone surgery?
   1. Yes
   2. No
   3. Unsure

Variable Code: SURGICUTI

Source: Gravel, Ronald, and Yves Béland. "The Canadian Community Health Survey: mental health and well-being." The Canadian Journal of Psychiatry 50.10 (2005): 573-579.

1. Rate your perception of surgical care
2. Excellent
3. Very Good
4. Fair
5. Poor
6. Very Poor

Variable Code: SURGICPER

Source: Gravel, Ronald, and Yves Béland. "The Canadian Community Health Survey: mental health and well-being." The Canadian Journal of Psychiatry 50.10 (2005): 573-579.

1. Rate your experience with surgical care:
   1. Positive
   2. Somewhat Positive
   3. Neutral
   4. Somewhat Negative
   5. Negative

Variable Code: SURGICEXP

Source: Gravel, Ronald, and Yves Béland. "The Canadian Community Health Survey: mental health and well-being." The Canadian Journal of Psychiatry 50.10 (2005): 573-579.

1. Have you encountered difficulty accessing surgery for any of the following reasons:
   1. Physician/Systemic Reasons
2. Waited too long for surgery
3. Difficulty getting an appointment with a surgeon
4. Waited too long for a diagnostic test
5. Still waiting for surgery
6. Difficulty getting a diagnosis
7. Waited too long for a hospital bed
8. Appointment cancelled/deferred
9. Service not available
   1. Patient Reasons
10. Deterioration of health
11. Cost
12. Difficulty acquiring transportation
13. Unable to leave house due to health
14. Personal/Family Responsibility
15. Language Barrier
    1. Other Reasons
       - Write in

Variable Code: SURGICBARR

Source: Gravel, Ronald, and Yves Béland. "The Canadian Community Health Survey: mental health and well-being." The Canadian Journal of Psychiatry 50.10 (2005): 573-579.

**Demographics**

1. How old are you?

___________ years old

- Don’t know / Not sure

Refused

Variable Code: AGE

Origin: Centers for Disease Control and Prevention (CDC). Behavioral Risk Factor Surveillance System Survey Questionnaire. Atlanta, Georgia: U.S. Department of Health and Human Services, Centers for Disease Control and Prevention, [2020]

2. Are you currently...?

1. Employed
2. Unemployed
3. Student
4. Retired

Variable Code: EMPLOYMENT

Origin: Centers for Disease Control and Prevention (CDC). Behavioral Risk Factor Surveillance System Survey Questionnaire. Atlanta, Georgia: U.S. Department of Health and Human Services, Centers for Disease Control and Prevention, [2020]

3. Are you…

1. Married
2. Divorced
3. Widowed
4. Separated
5. Never married

Variable Code: MARITAL

Origin: Centers for Disease Control and Prevention (CDC). Behavioral Risk Factor Surveillance System Survey Questionnaire. Atlanta, Georgia: U.S. Department of Health and Human Services, Centers for Disease Control and Prevention, [2020]

4.Where do you live?

1. Irbid
2. Ajloun
3. Jerash
4. Mafraq
5. Balqa
6. Amman
7. Zarqa
8. Madaba
9. Karak
10. Tafila
11. Ma'an
12. Aqaba

Variable Code: ORIGIN
