# Supplementary figures and images for: Healthcare access and barriers in Jordan: Insights from a Nationwide Survey
Source: PLoS One. 2026 Mar 24;21(3):e0345456. doi: 10.1371/journal.pone.0345456 (PMC13012521; doi:10.1371/journal.pone.0345456)

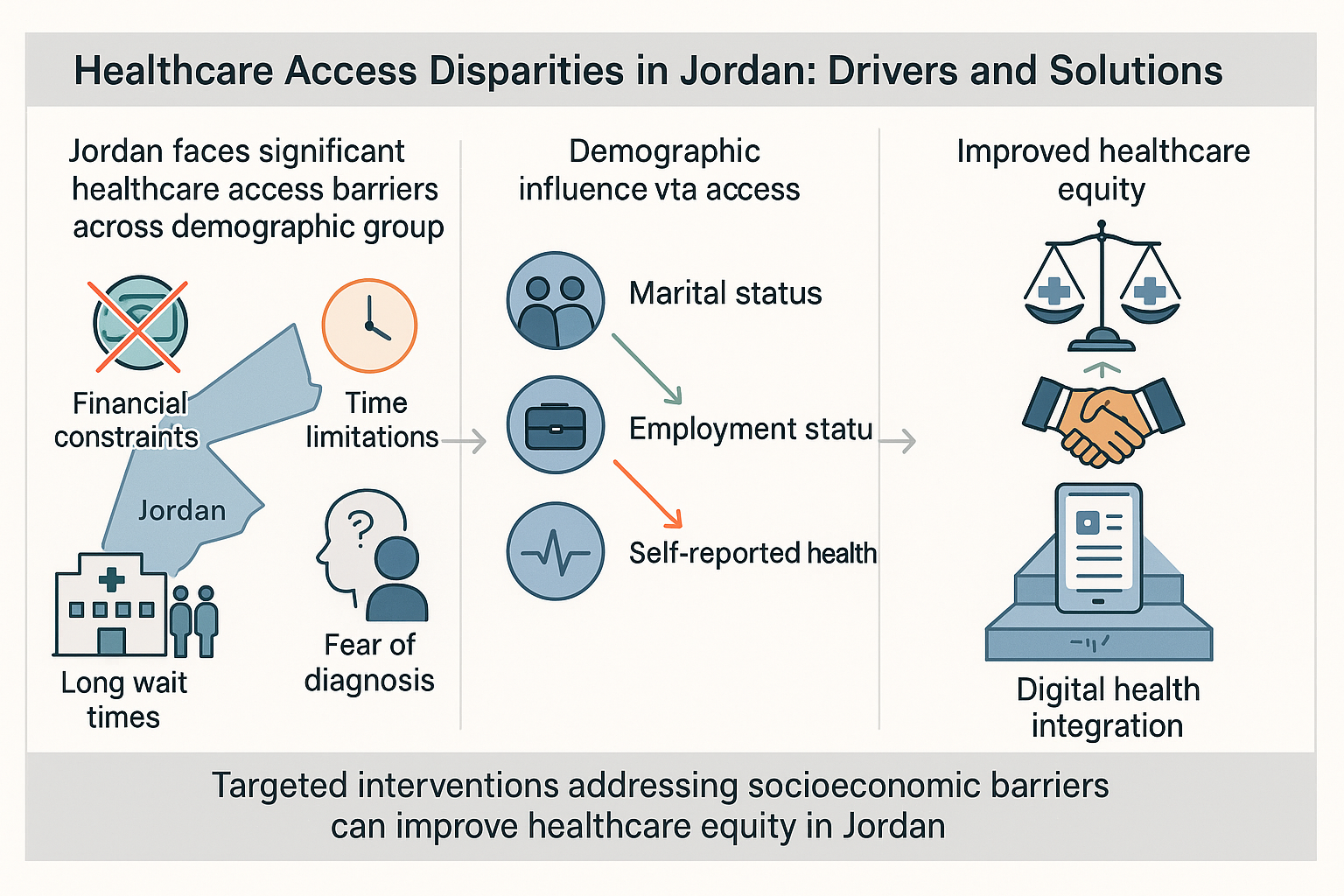

Supplement: S1 Fig — (PNG) [file pone.0345456.s002.png]
